# Alteration of Antioxidant Enzymes and Associated Genes Induced by Grape Seed Extracts in the Primary Muscle Cells of Goats *In Vitro*

**DOI:** 10.1371/journal.pone.0107670

**Published:** 2014-09-19

**Authors:** Tan Yang, Xiaomin Li, Wang Zhu, Cheng Chen, Zhihong Sun, Zhiliang Tan, Jinghe Kang

**Affiliations:** 1 Key Laboratory for Bio-Feed and Molecular Nutrition, Southwest University, Chongqing, P. R. China; 2 Key Laboratory of Agro-Ecological Processes in Subtropical Region, Hunan Research Center of Livestock & Poultry Sciences, South-Central Experimental Station of Animal Nutrition and Feed Science in Ministry of Agriculture, Institute of Subtropical Agriculture, The Chinese Academy of Sciences, Changsha, Hunan, P.R. China; University of Catania, Italy

## Abstract

This study was conducted to investigate how the activity and expression of certain paramount antioxidant enzymes respond to grape seed extract (GSE) addition in primary muscle cells of goats. Gluteal primary muscle cells (PMCs) isolated from a 3-week old goat were cultivated as an unstressed cell model, or they were exposed to 100 µM H_2_O_2_ to establish a H_2_O_2_-stimulated cell model. The activities of catalase (CAT), superoxide dismutases (SOD) and glutathione peroxidases (GPx) in combination with other relevant antioxidant indexes [i.e., reduced glutathione (GSH) and total antioxidant capacity (TAOC)] in response to GSE addition were tested in the unstressed and H_2_O_2_-stimulated cell models, and the relative mRNA levels of the CAT, GuZu-SOD, and GPx-1 genes were measured by qPCR. In unstressed PMCs, GSE addition at the dose of 10 µg/ml strikingly attenuated the expression levels of CAT and CuZn-SOD as well as the corresponding enzyme activities. By contrast, in cells pretreated with 100 µM H_2_O_2_, the expression and activity levels of these two antioxidant enzymes were enhanced by GSE addition at 10 µg/ml. GSE addition promoted GPx activity in both unstressed and stressed PMCs, while the expression of the GPx 1 gene displayed partial divergence with GPx activity, which was mitigated by GSE addition at 10 µg/ml in unstressed PMCs. GSH remained comparatively stable except for GSE addition to H_2_O_2_-stimulated PMCs at 60 µg/ml, in which a dramatic depletion of GSH occurred. Moreover, GSE addition enhanced TAOC in unstressed (but not H_2_O_2_-stimulated) PMCs. GSE addition exerted a bidirectional modulating effect on the mRNA levels and activities of CAT and SOD in unstressed and stressed PMCs at a moderate dose, and it only exhibited a unidirectional effect on the promotion of GPx activity, reflecting its potential to improve antioxidant protection in ruminants.

## Introduction

Grape seed extract (GSE) has been well-documented for its effect on antioxidation, and it has been extensively used as therapy in substantial diseases of diverse organs and tissues such as asthma, cataracts, cardiovascular diseases, small intestinal mucositis and nephropathy [1]–[5]. Although the underlying mechanisms relevant to the pathogenesis of these diseases have not yet been completely clarified, reactive oxygen species (ROS) are purportedly an important contributor, and the therapeutic function of GSE is tightly linked to its antioxidant effectiveness [1]–[5]. Recently, accumulated evidence has demonstrated that replenishing GSE is a feasible way of alleviating the negative effects of ROS within skeletal muscle. It has been found that skeletal muscle cells frequently become the targets of ROS under assorted conditions [6]–[10]. ROS-mediated oxidative damage and signal interruption both cause cell dysfunction to some extent [11], [12]. It has been observed that the exposure of myotubes to ROS (i.e., H_2_O_2_ and Doxorubicin) results in muscle protein wasting and fiber atrophy [13]–[15]. In addition, scores of studies have shown the adverse effects of ROS on skeletal muscle in rats fed high-carbohydrate/high-fat diets [12], [16]. However, subsequent testing has indicated that GSE has the ability to alleviate ROS damage by acting as an effective antioxidant [17].

The antioxidant function of GSE is mediated by a vast array of functional components with a phenolic nature such as monomeric flavanols; dimeric, trimeric and polymeric procyanidins; and phenolic acids [4], [18]. These phenolic compounds are endowed with the capacity to scavenge singlet oxygen and peroxyl radicals via the reduction of multiple O–H bonds [19]. The effectiveness of phenolic compounds as free radical scavengers was proven to greatly exceed those of vitamins E and C, the major recognized antioxidants in biological systems [20]. However, it is noteworthy that, in the scope of existing GSE research, the beneficial effects of GSE against oxidative radicals are mainly of a chemical nature. Relatively few studies have addressed whether there is a biological gateway for GSE in relation to the prevention of oxidative challenge.

In fact, phenolic and polyphenolic compounds that act as natural antioxidants are primarily present in and isolated from fruits, some vegetables, tea and herbs [18], [21]. Temporarily ignoring the differences between grapeseeds and other botanicals with diverse molecular structures or different amounts of similar polyphenols, it has been found that the protective effect of genistein, an isoflavone mostly found in legumes, against diabetes-induced renal damage is partly dependent on the improvement of glutathione peroxidase (GPx) and superoxide dismutase (SOD) [22]. Our previous study also found that tea catechins can regulate antioxidant enzymes to alleviate H_2_O_2_-induced damage in goat skeletal muscle cells in vitro [23].

We hypothesized that GSE most likely exerts its antioxidant function by influencing the gene expression levels and production of some or all antioxidative enzymes in various tissues, although chemoprotection is not necessarily exclusively dependent on this. In this study, we aimed to test the putative antioxidative function of GSE from a fresh perspective in ruminants by examining the effect of GSE on antioxidant enzyme activity and evaluating the influence of GSE at the gene level on corresponding enzymes in primary muscle cells of goats.

## Materials and Methods

### Ethics Statement

This study was carried out in strict accordance with the recommendations in the Animal Care and Use Guidelines of the Institute of Subtropical Agriculture (ISA), Chinese Academy of Sciences. The protocol was approved by the Animal Care Committee on the Ethics of Animal Experiments of ISA. All surgery was performed under sodium pentobarbital anesthesia, and all efforts were made to minimize suffering.

### Extracts and Reagents

Commercially available dried and powdered GSE obtained from Tarac Technologies (Nuriootpa, South Australia) contained 5.01% (+)-catechin, 4.78% (−)-epicatechin, 2.35% (−)-epigallocatechin, 14.1% dimeric proanthocyanidin, 11.60% trimeric proanthocyanidins, 7.69% tetrameric proanthocyanidins and 40.0% polymeric proanthocyanidins. Dulbecco's Modified Eagle Medium (DMEM), fetal bovine serum, penicillin, and streptomycin were purchased from Invitrogen (Rockville, MD). Antioxidant detection kits were ordered from Nanjing Jiancheng Bioengineering Institute (Nanjing, China) and Beyotime Institute of Biotechnology (Shanghai China). Unless otherwise specified, all other chemicals were from Sigma (St. Louis, MO).

### Cell Isolation, Culture and Treatment

The primary muscle cells (PMCs) used in our experiments were isolated from the gluteal muscles of one Liu Yang black Goat (a local breed, south of China) according the method of Zhong et al (who modified the existing method of Lynge et al) [23], [24]. The experimental goat was carefully chosen from 3-week old weaned healthy goats. The PMCs were incubated in DMEM medium containing 10% FBS and a 1% mixture of penicillin and streptomycin (10 units/mL and 1 mg/mL, respectively) at 37°C under an atmosphere of 5% CO_2_/95% air. The growing medium was changed every two days. At 80–90% confluence, the PMCs were washed with phosphate buffered saline (PBS) once and subcultured with 0.25% trypsin–EDTA solution. Without a specific indication, activated PMCs were used at the age of passage 4–8 and starved to FBS for 24 h before any treatment.

Before the formal treatment, PMCs were exposed to a stepwise series of increasing concentrations of H_2_O_2_ to induce oxidative stress and oxidative damage. After examining cell activity, 100 µM H_2_O_2_ was determined as the optimum concentration in the later formal experiment. Likewise, a dose-response study based on a wide range of GSE concentrations from 0.1 to 60 µg/ml medium was performed in the absence and presence of H_2_O_2_ preincubation. Based on cell viability, GSE levels of 1, 10 and 60 µg/ml were chosen as the ultimate addition levels in this trial, representing mild, moderate and excessive doses, respectively.

To characterize the effects of GSE on PMCs in un-stressed and stressed conditions, two PMC models were separately established as follows: cells were cultured in medium at the GSE levels of 1, 10 and 60 µg/ml for 24 h as the un-stressed cell model; a stressed cell model was established wherein cells were induced by accretion with 100 µM H_2_O_2_ in the medium for 1 h. It should be noted here, after incubation for 1 h with 100 µM H_2_O_2_, the cells were washed twice with PBS in order to remove H_2_O_2_ and to avoid it reacting with GSE directly, followed by the protocol of un-stressed cell model. Untreated cells serving as a control were also run in parallel and subjected to the same medium as the PMC models.

### Cell Viability Tests

Cells were seeded in a 96-well plate (1×10^4^ cells/well), and after the above-mentioned treatment, they were washed twice with PBS to eliminate interference with the subsequent assay. Cell viability was assessed using the commercial cell counting kit-8 (Dojindo, China) according to the manufacturer's instructions.

### Microscopic Analysis

Cells were plated onto 34.8-mm dishes at a density of 2×10^6^ cells and cultured to 70–80% confluence at 37°C. Then, they were incubated under different conditions, after which cell morphology was observed using a microscope (CX31, Olympus, Japan).

### Total Antioxidant Capacity (TAOC) and Reduced Glutathione (GSH) Measurement

The TAOC in supernatants of cells lysed was measured using the azino-diethyl-benzthiazoline sulfate (ABTS) method. In this assay, incubation of ABTS with H_2_O_2_ and a peroxidase (metmyoglobin) results in the production of the blue-green radical cation ABTS+. Antioxidants in the sample suppress this color production proportionally to their concentration [25]. The system was standardized using Trolox, a water-soluble vitamin E analogue. The results were expressed as mmol Trolox equivalent/protein concentration of plasma supernatant of lysed cells. Supernatants of lysed cells were analyzed for GSH using the Glutathione Quantification Kit (Jianchen, Co., Nanjing, China) according to the manufacturer's instructions. This kit employs a fundamental reaction in which 5,5′-dithiobis-2-nitrobenzoic acid (DTNB) and GSH react to generate 2-nitro-5-thiobenzoic acid and glutathione disulfide (GSSG). Because 2-nitro-5-thiobenzoic acid is a yellow product, the GSH concentration in a sample can be detected at 405 nm.

### Antioxidant Enzyme Activity Assay

Briefly, cells were harvested in a lysate buffer containing 0.1% Triton X-100, then centrifuged to remove the supernatant for the assay [13]. Catalase (CAT) activity in the cell homogenates was determined using a CAT analysis kit purchased from Beyotime Bio-Corporation (Shanghai, China). As per the manufacturer's instructions, samples were placed in a cuvette that received excess hydrogen peroxide for decomposition by CAT in our samples for an exact time between 1 to 5 min, and the remaining hydrogen peroxide coupled with a substrate was treated with peroxidase to generate a red product, N-4-antipyryl-3-chloro-5-sulfonate-p-benzoquinonemonoimine, which absorbs maximally at 520 nm. In this way, CAT activity was determined by measuring the decomposition of hydrogen peroxide spectrophotometrically (Bio-RAD680, Bio-rad Co., USA). The activity of SOD was measured in 96-well microplates using the WST-1 method. WST-1, a 2-(4-iodophenyl)-3-(4-nitrophenyl)-5-(2,4-disulfophenyl)-2H-tetrazolium, monosodium salt, exhibits very low background absorbance and is efficiently reduced by superoxide to a stable water-soluble formazan with high molar absorptivity. As the superoxide production from xanthine oxidase in the SOD kit (from Beyotime Bio-Corporation, Shanghai, China) could be cut down by SOD in cell lysate suspensions, the optical density of formazan was measured at 450 nm. The activity of GPx was estimated using the commercially available GPx assay kit from Beyotime Bio-Corporation (Shanghai, China). The GPx activity was measured indirectly by a coupled reaction with glutathione reductase. Oxidized glutathione produced via the reduction of hydroperoxide by GPx was recycled to its reduced form by the oxidation of triphosphopyridine nucleotide reduced tetrasodium salt to nicotinamide adenine dinucleotide phosphate (NADPH to NADP+), which was accompanied by a decrease in absorbance at 340 nm [26].

### RNA Isolation and Real-Time RT-PCR

A total of 1 ml of ice-cold TRIzol reagent (TaKaRa, Japan) was added to cell monolayers. Extraction was carried out according to the manufacturer's instructions. The integrity of the isolated RNA was checked using agarose gel electrophoresis (1%). The RNA concentration was calculated from the absorbance at 260 nm. Protein contamination was further assessed by spectrophotometric determination of the absorbance 260 nm to 280 nm ratio, and only samples with a ratio between 1.8 and 2.1 were used in further experiments. To remove traces of chromosomal DNA, 1000 ng of total RNA was treated with RNase-free DNase I (TaKaRa, Japan) for 5 min at 37°C. Quantitative real-time PCR (qRT-PCR) was performed using the SYBR Premix Ex TaqTM Kit (TaKaRa, Japan) on the Applied Biosystems Prism 7900 HT sequence detection system (Applied Biosystems, Foster, CA). The cDNA was diluted fourfold before equal amounts were added to duplicate qRT-PCR reactions. The tested genes and their sequences designed according to Zhong et al. [23] are listed in Table 1. The thermal profile for all reactions was 30 sec at 95°C, then 40 cycles of denaturation at 95°C for 5 s, annealing at 60°C for 30 s and extension at 72°C for 30 s. At the end of each cycle, the fluorescence monitoring was for 10 s. Each reaction was completed with a melting curve analysis to ensure the specificity of the reaction. Gene expression was quantified using real-time qPCR analyzer software by Eppendorf. The relative expression levels of mRNA species were determined using the comparative Ct method. β-actin was selected as a reference gene. The expression ratio (R) of treatment to control cells was calculated using the following equations: R = 2^−ΔΔCt^; and ΔΔCt  =  (Ct_target gene_ − Ct_β-actin_) treatment − (Ct_target gene_ − Ct_β-actin_) control. The reactions for each sample were performed in quadruplicate.

**Table 1:** Sequences of primers (forward, for; reverse, rev), sizes of primers, and sizes of real-time quantitative PCR products.

Target gene	Primer (5′- 3′)	Primer size (bp)	Product size (bp)
CAT, for	TGGGACCCAACTATCTCCAG	20	207
CAT, rev	AAGTGGGTCCTGTGTTCCAG	20	
CuZn-SOD, for	TGCAGGCCCTCACTTTAATC	20	216
CuZn-SOD, rev	CTGCCCAAGTCATCTGGTTT	20	
GPx 1, for	ACATTGAAACCCTGCTGTCC	20	178
GPx 1, rev	TCATGAGGAGCTGTGGTCTG	20	
β-actin, for	CCAACCGTGAGAAGATGACC	20	201
β-actin, rev	CGCTCCGTGAGAATCTTCAT	20	

CAT  =  catalase; CuZn-SOD  =  CuZn superoxide dismutase; GPx  =  glutathione peroxidase.

### Statistical Analyses

Data are presented as the mean ± SEM for all determinations. Significant differences were analyzed by 1-way ANOVA of SAS or Mann Whitney tests as appropriate. A P value <0.05 was considered significant.

## Results

**Figure 1:**
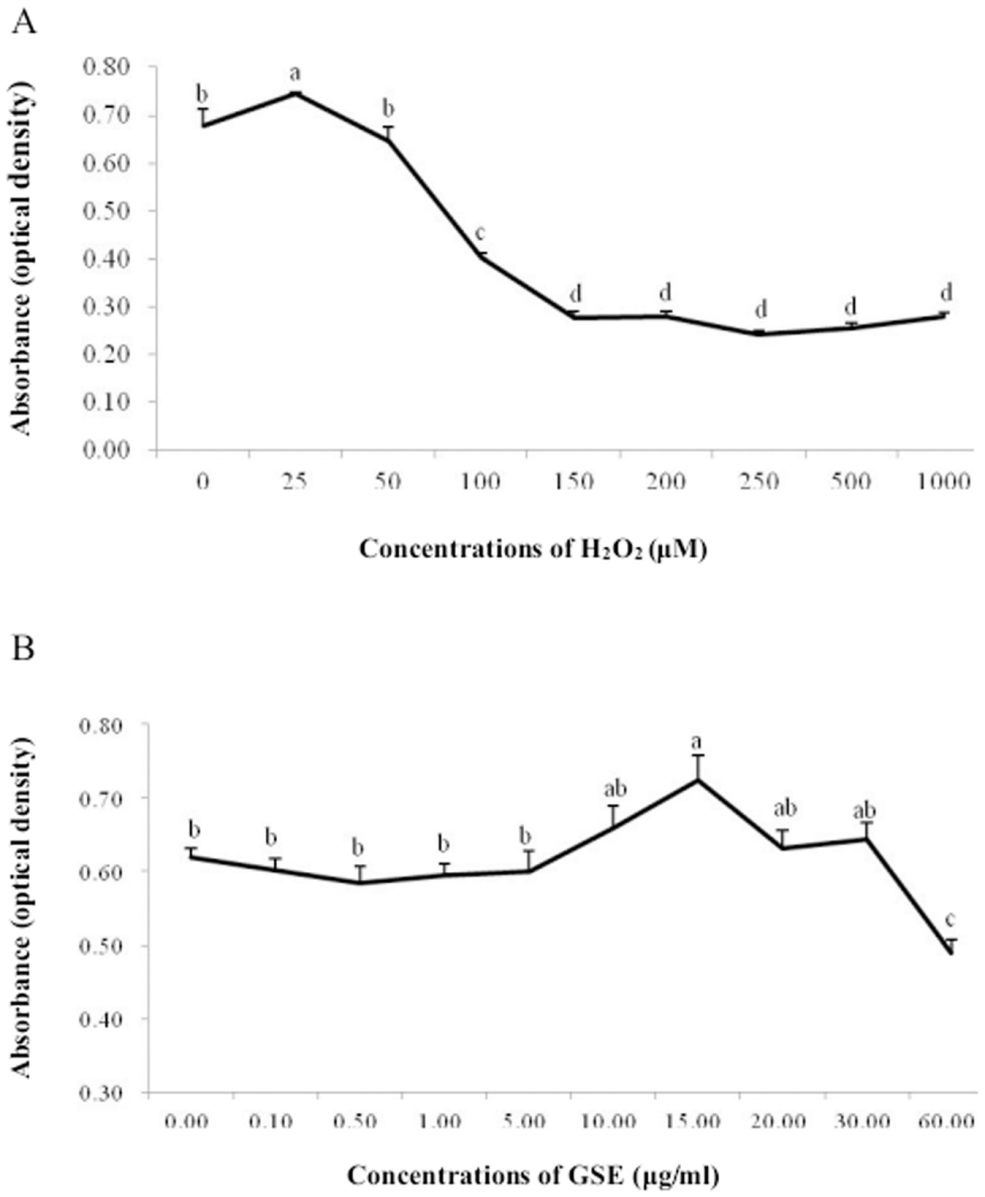
Alteration of primary muscle cells (PMCs) viability after incubation with various concentrations of H_2_O_2_ for 1 h (A), or GSE for 24 h (B). The results are presented as the means ± SEM (n = 12). Mean values with different letters differ (*P*<0.05).

A sharp dose-dependent decrease of PMC viability is revealed in Figure 1A. When the concentration of H_2_O_2_ surpassed 25 µM, this decline continued until the H_2_O_2_ concentration reached 150 µM. Subsequently, the cell viability remained comparatively stable but at a low level, even with H_2_O_2_ up to 1000 µM. The cell viability curve indicated that the PMCs were sensitive to H_2_O_2_ in the range from 25 to 150 µM. We thereby chose 100 µM H_2_O_2_ as the optimal concentration for inducing oxidative stress in PMCs in the subsequent formal trial, in part because that is in the sensitive range, and a 50% disruption of cells compared to the control was observed. Figure 1B depicts PMC viability after incubation with diverse concentrations of GSE for 24 h. Cell viability was unaffected until the GSE concentration reached 10 µg/ml. GSE at 15 µg/ml resulted in a marked increase of cell viability, but the increasing trend did not continue with higher GSE concentrations. Subsequently, cell viability fell into a recession and reached its minimum at the level of 60 µg/ml.

**Figure 2:**
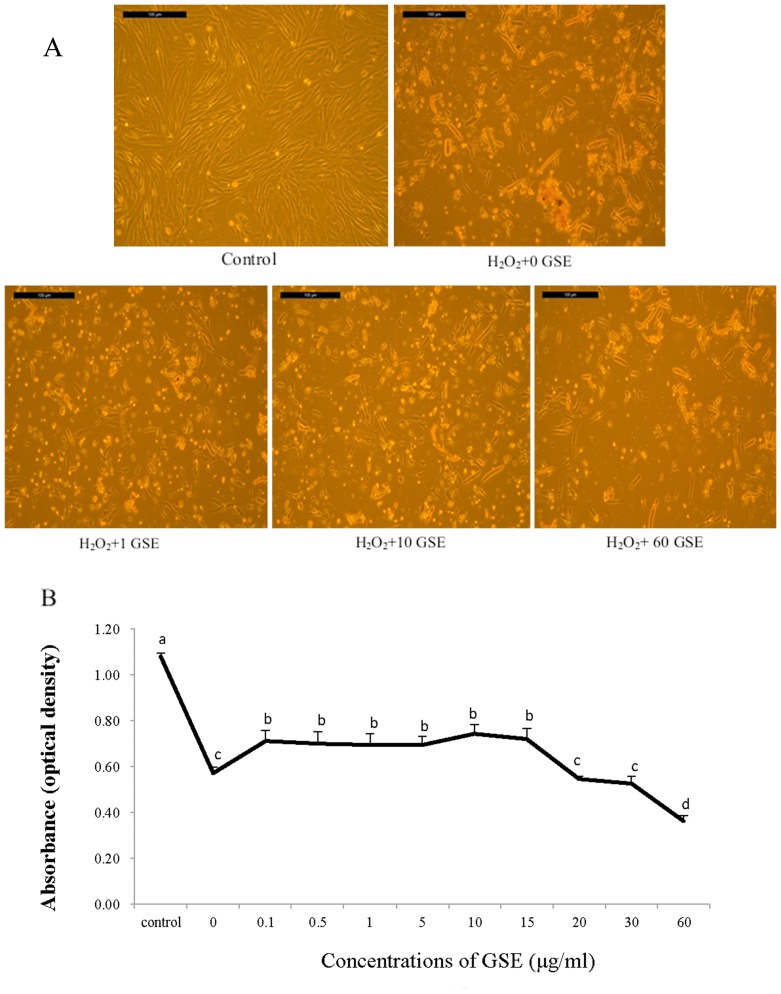
Representative photographs (A) and viability (B) of primary muscle cells (PMCs) pretreated with 100 µM H_2_O_2_ for 1 h followed by incubation with various concentrations of GSE for 24 h. The values are represented as the means ± SEM (n = 12). Mean values with different letters differ (*P*<0.05).

Figure 2A shows photographs of PMCs (control) and PMCs subjected to the insult of 100 µM H_2_O_2_ for 1 h and then incubated with 0, 1, 10 or 60 µg GSE/ml for 24 h. Compared with the control, PMCs treated only with H_2_O_2_ had obvious swelling, showed a dramatic increase in floating cells (non-adhering cells), and the aggregation of floating cells even appeared. For H_2_O_2_-pretreated PMCs supplemented with GSE at 1 or 10 ug/ml, the adhering cells' morphology remained virtually unchanged, although there was still a significant increase of floating cells compared with the control. As the GSE addition level reached 60 ug/ml for H_2_O_2_-pretreated PMCs, the amount of cells was visually scarce, even compared with PMCs treated with H_2_O_2_ alone. Figure 2B reflects the viability of PMCs that initially suffered from the insult of 100 µM H_2_O_2_ for 1 h then were incubated with various GSE concentrations for 24 h. The viability of PMCs subjected to H_2_O_2_ challenge was less (P<0.05) than that of the control. However, subsequent GSE supplementation of 0.1–15 ug/ml successfully mitigated the inhibitory effect of H_2_O_2_ on cell viability. When GSE addition surpassed 15 µg/ml, cell viability markedly declined, and it dropped to its lowest value at the GSE level of 60 ug/ml.

**Figure 3:**
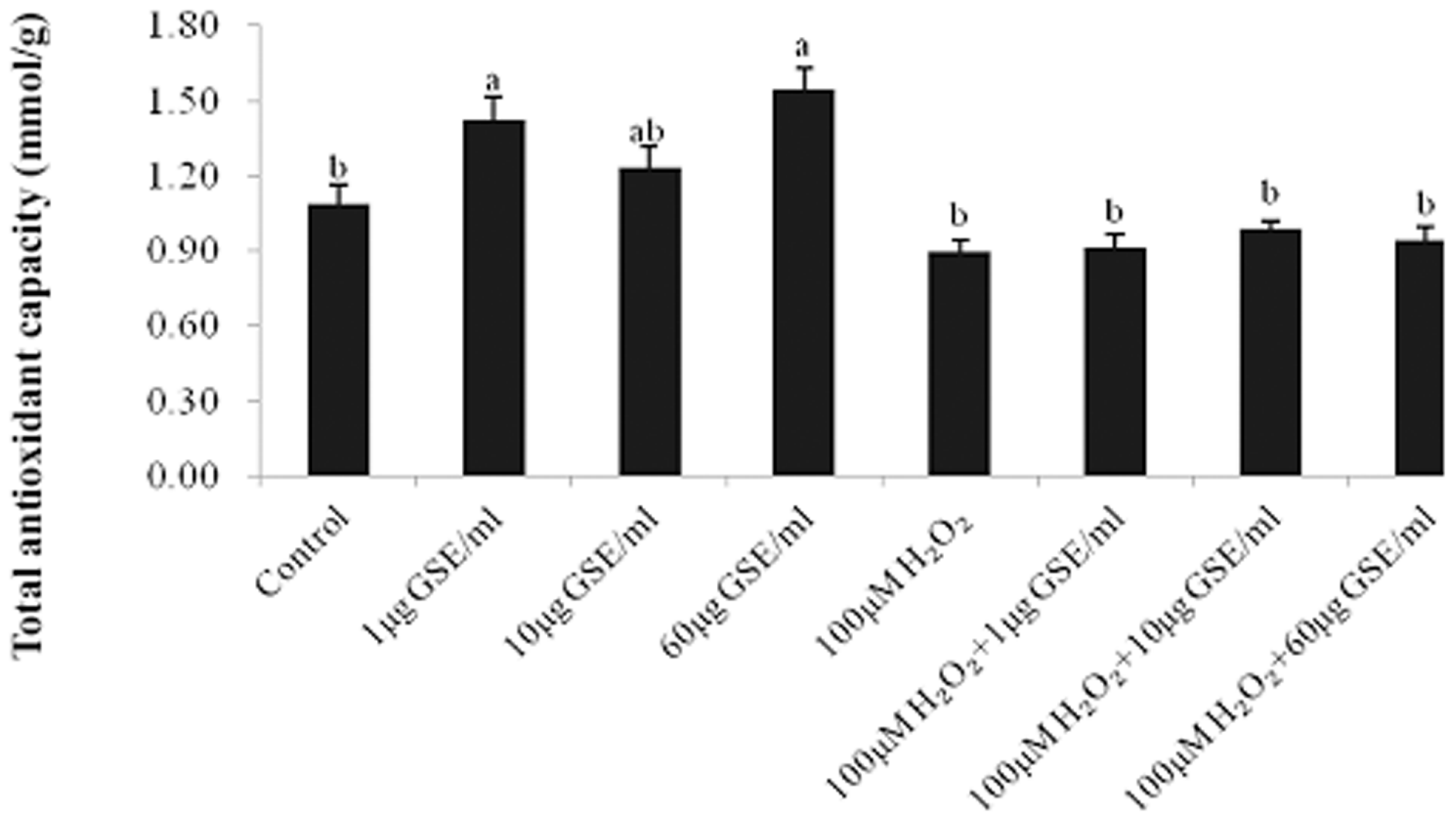
Alteration of total antioxidation capability (TAOC) after incubation of primary muscle cells (PMCs) with various concentrations of GSE for 24 h with or without H_2_O_2_ pretreatment. Bars represent the mean ± SEM (n = 4). Bars not sharing a common letter differ (*P*<0.05).

GSE addition at 1 and 60 µg/ml led to a significant increase of TAOC in un-stressed PMCs compared with the control (Figure 3). However, TAOC was not affected by GSE addition to cells pretreated with H_2_O_2_. GSE addition did not affect the GSH content in un-stressed PMCs with an average value of 49.2±0.9 nmol/mg protein Treatment with H_2_O_2_ led to a significant depletion of GSH (40.6±3.2 nmol/mg protein). Following GSE addition at levels of 1 and 10 µg/ml, GSH content did not recover, with respective values of 41.2±2.8 and 42.0±3.4 nmol/mg protein, and it even dramatically decreased to 32.2±1.9 nmol/mg protein as the GSE dose reached 60 µg/ml.

**Figure 4:**
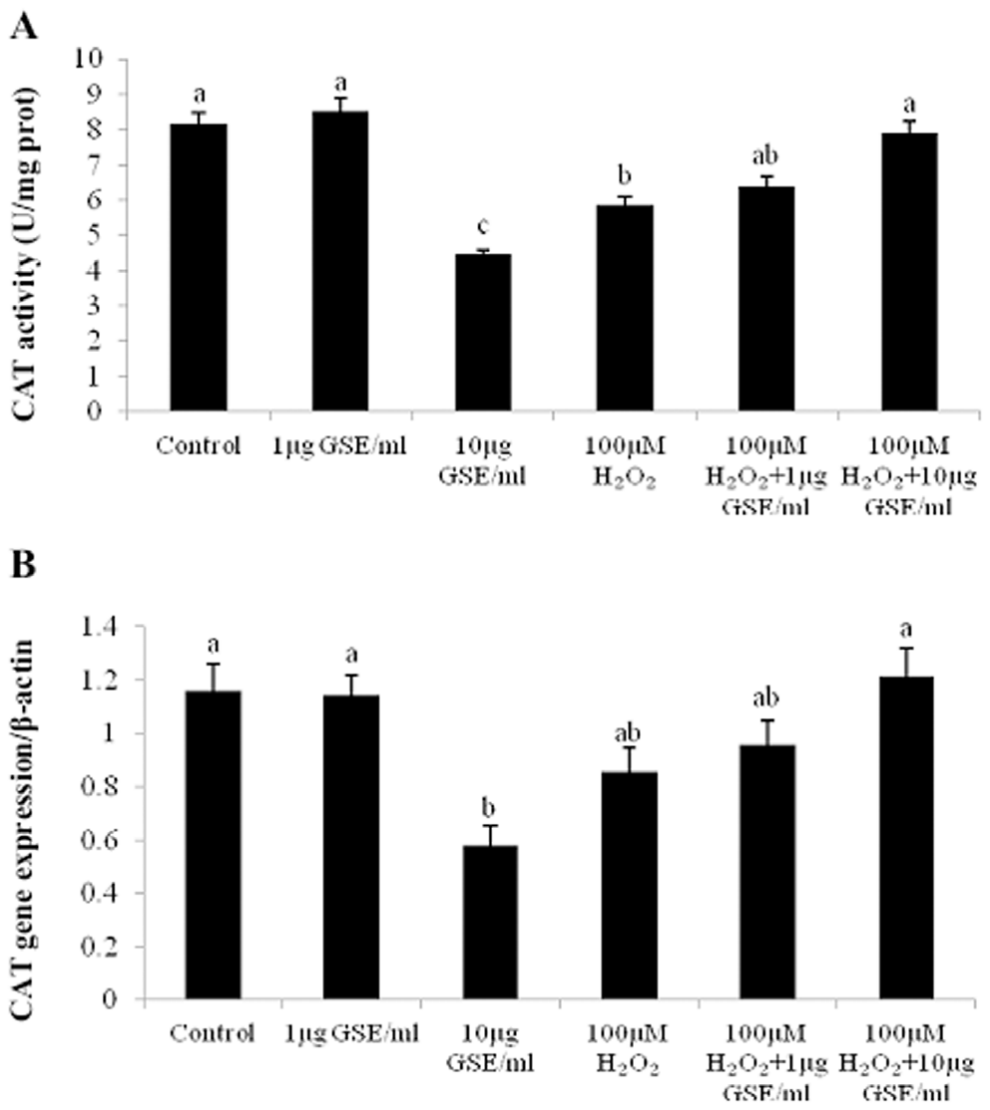
Effects of GSE addition on catalase (CAT) activity (A) and the relative CAT mRNA levels (B) of primary muscle cells (PMCs) pretreated with or without H_2_O_2_. Bars represent the mean ± SEM (n = 5) (A). Bars represent the mean ± SEM (n = 4) (B). Bars not sharing a common letter differ (*P*<0.05).

Figure 4 shows the effects of GSE addition on the activity and relative mRNA level of CAT in PMCs under un-stressed and stressed conditions. With un-stressed PMCs, GSE addition of 10 µg/ml decreased (P<0.05) CAT activity in comparison with the control and mild-dose groups (Figure 4A). CAT activity was reduced by approximately 45% at the GSE level of 10 µg/ml compared to the control. However, in stressed cells, GSE addition at 10 µg/ml conversely increased (P<0.05) the CAT activity compared to that without GSE addition. Concerning the significant drop of cell viability at the level of 60 µg GSE/ml (Figure. 2), the death of a great number of cells altered the culture environment of the surviving cells. Because it would be no longer correct to compare the enzyme activities and mRNA levels, these data at the dose of 60 µg GSE/ml were thereby not shown in the relevant figures. In PCR analysis, the mRNA expression level of the CAT gene was unaffected by GSE addition at 1 µg/ml, but it was down-regulated at 10 µg/ml in un-stressed PMCs, and thus corresponded well to the alterations of CAT activity. Following H_2_O_2_ treatment, the mRNA level of the CAT gene was less (P<0.05) than that of the control. Subsequent addition of GSE increased the CAT mRNA level only marginally.

**Figure 5:**
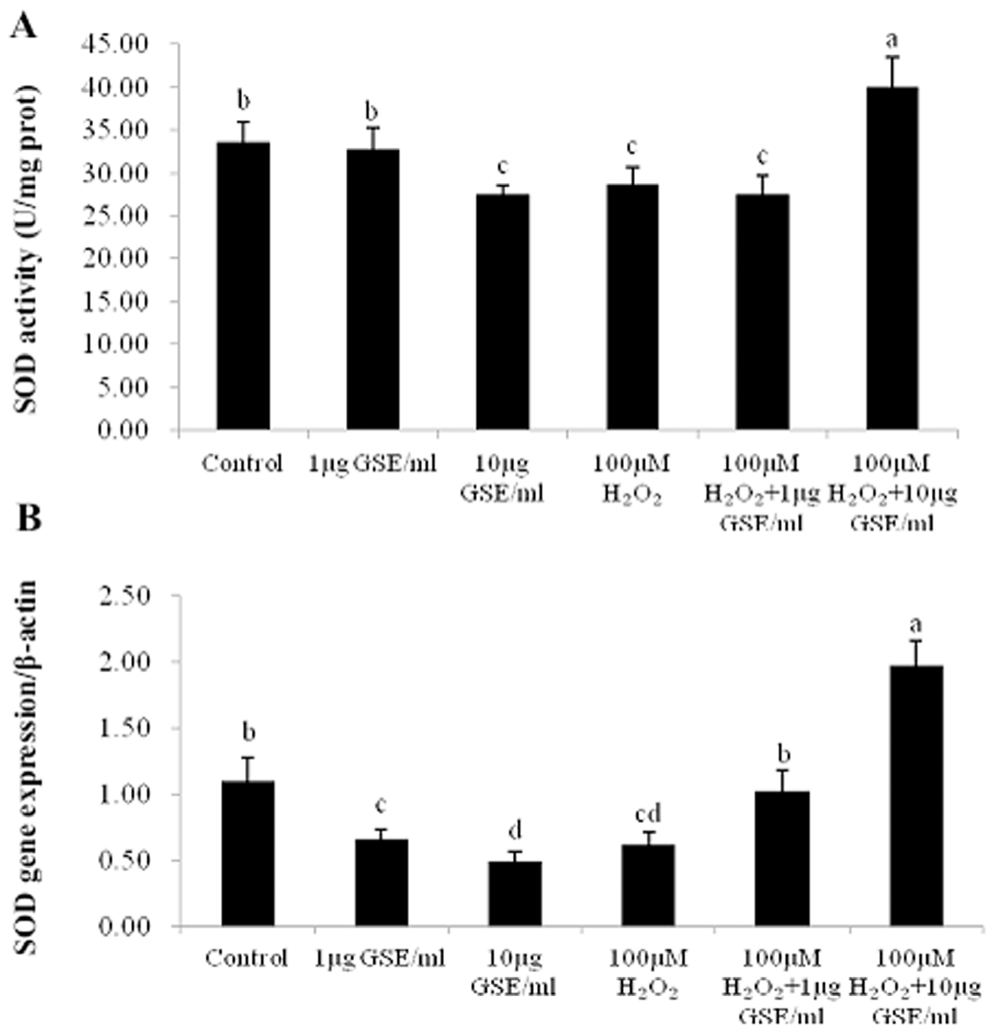
Effects of GSE addition on SOD activity (A) and the relative CuZn-SOD mRNA levels (B) of primary muscle cells (PMCs) pretreated with or without H_2_O_2_. Bars represent the mean ± SEM (n = 5) (A). Bars represent the mean ± SEM (n = 4) (B). Bars not sharing a common letter differ (*P*<0.05).

Figure 5 illustrates effects of GSE addition on another antioxidant enzyme, SOD, in terms of enzyme activity and CuZn-SOD gene expression. Adding GSE at 10 µg/ml weakened (P<0.05) the SOD activity and the CuZn-SOD mRNA level relative to the control. With respect to the control, the SOD activity and CuZn-SOD mRNA level were inhibited (P<0.05) by 100 µM H_2_O_2_ treatment alone; however, these values rebounded following the addition of GSE at the dose of 1 or 10 ug/ml.

**Figure 6:**
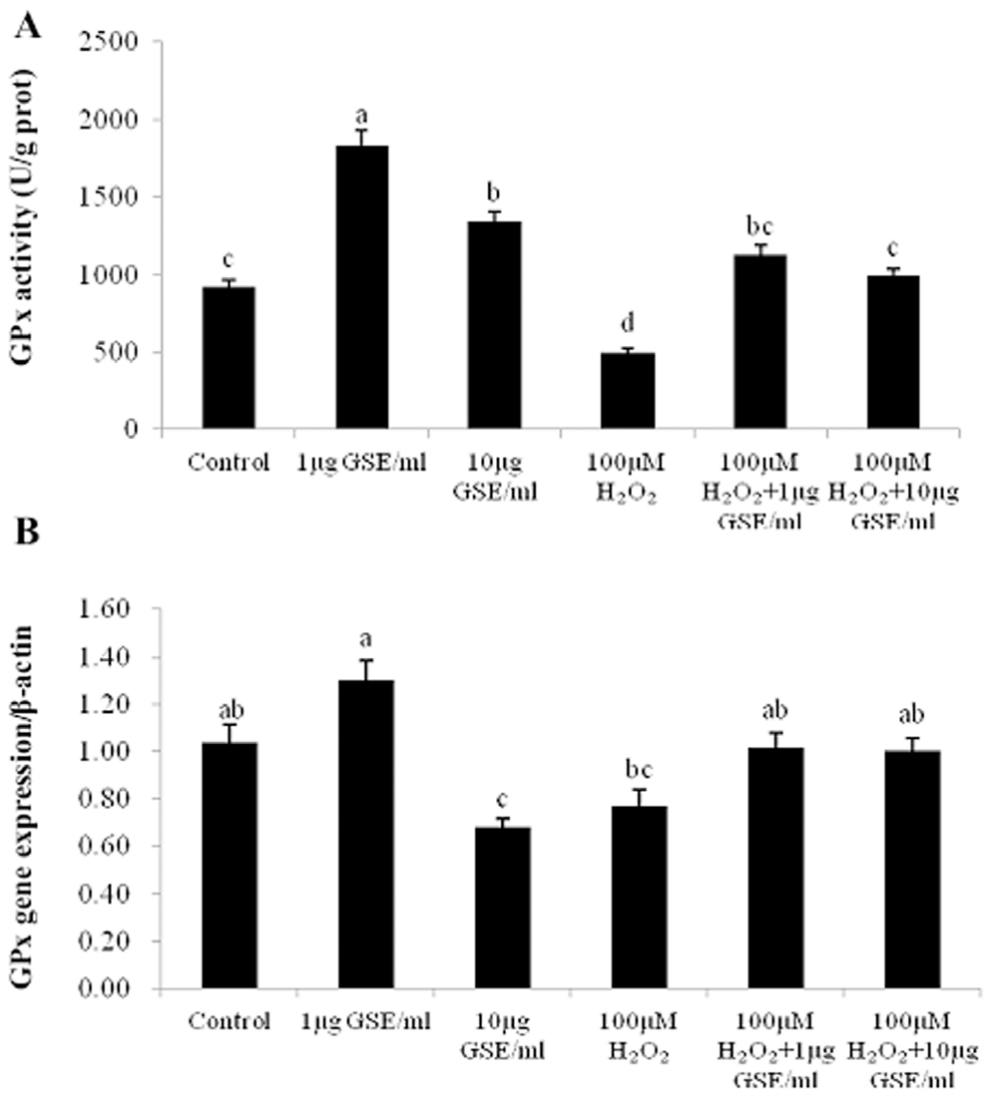
Effects of GSE addition on GPx activity (A) and the relative GPx 1 mRNA levels (B) of primary muscle cells (PMCs) pretreated with or without H_2_O_2_. Bars represent the mean ± SEM (n = 5) (A). Bars represent the mean ± SEM (n = 4) (B). Bars not sharing a common letter differ (*P*<0.05).

GSE supplementation at the dose of 1 or 10 µg/ml, regardless of whether the PMCs were un-stressed or stressed, promoted (P<0.05) GPx activity compared with the control (Figure 6). In regard to the mRNA level of GPx 1, there was a numerical increase in the GPx 1 mRNA level of un-stressed PMCs at the GSE dose of 1 µg/ml, whereas a moderate dose of GSE (10 µg/ml) caused a remarkable reduction (P<0.05). Exposure to H_2_O_2_ for only 1 h numerically reduced the GPx 1 mRNA expression of PMCs when compared to the control. GSE addition marginally increased GPx 1 mRNA levels of PMCs in contrast with those of H_2_O_2_-exposed cells.

## Discussion

Excess production of ROS has been widely recognized to pose huge threats to the health of humans and animals [22]. It triggers oxidative damage and dysfunction in various cell types, including skeletal muscle cells, and it is strongly linked to the pathogenesis of a broad spectrum of diseases [9], [10]. GSE has been extensively reported to effectively scavenge ROS and protect against ROS-associated damage. Acute skeletal muscle damage in rats results in skeletal muscle fiber disruption, oxidative stress and inflammation, while the administration of a grape seed-derived proanthocyanidolic oligomer before and after the injury facilitates faster effective regeneration of the injured skeletal muscle [27]. Similarly, in the current experiment, direct exposure of PMCs to H_2_O_2_ led to a serious impairment as assessed by cell viability and morphological observations. However, with the addition of GSE at the proper dose, the viability of H_2_O_2_-pretreated PMCs clearly recovered, indicating that GSE confers antioxidant protection to goat PMCs.

TAOC is a valuable biomarker now widely used in analyses of serum, feedstuffs and biological tissues [28]. It is generally believed that cells and tissues of the body become more prone to develop dysfunction when antioxidant defenses are weakened. GSE, which serves as an important natural antioxidant, has been proven to improve TOAC in several experiments. Cyclosporine A is an immunosuppressive drug that has been proposed to expedite the generation of ROS in cardiac muscle cells based on significant increases in oxidative stress index and malondialdehyde. Feeding rats Cyclosporine A together with GSE not only mitigates the levels of oxidative stress and malondialdehyde, it also elevates cardiac TOAC [29]. In the present study GSE addition dramatically raised the TAOC of un-stressed PMCs but not of stressed PMCs. Hence, the maintenance of adequate antioxidant levels is essential to preventing or merely managing a great number of cell dysfunctions and disease conditions [28].

Previously, the antioxidant properties of GSE were basically attributed to GSE's intrinsic reducing capabilities [30], [31]. However, the present study provides some novel evidence that GSE has the ability to modulate the activity and gene expression levels of relevant antioxidant enzymes. We observed severe declines in CAT and SOD activity in un-stressed PMCs incubated with a moderate dose of GSE, and corresponding changes in the relative mRNA levels of CAT and CuZn-SOD genes occurred. Activation of ERK1/2 has been shown to negatively correlate with CAT activity [32]. Additionally, it has been reported that GSE treatment of human colon carcinoma HT29 cells results in strong dose- and time-dependent phosphorylation of ERK1/2 [33]. Presumably, the ERK1/2 signaling pathway provides an effective approach for GSE to attenuate CAT expression and activity in unstressed PMCs. However, striking increases in CAT and SOD activity have been observed in H9C2 (rat heart cell line) cells incubated with catechin or proanthocyanidin B4, two important components of GSE. The inconsistency in comparison with our results is putatively due to the different cells used and some discrepancies in the GSE agents [34].

Interestingly, in H_2_O_2_-pretreated PMCs, the activities of both CAT and SOD plus the mRNA level of the CuZn-SOD gene were all positively influenced by a moderate dose of GSE. Previous research found that diabetic rats show increased oxidative stress in the body and down-regulated CAT protein expression in the aorta, but CAT protein expression was up-regulated after feeding with proanthocyanidin extracted from grapeseeds [35]. In addition, although decreased CAT and SOD activities have been observed in ischemically injured rat myocardium (which induces excessive ROS generation), the administration of proanthocyanidin derived from grapeseeds effectively increases the activities of CAT and SOD [36]. Peroxisomal proliferator-activated receptors (PPARs) have been proven to suppress ROS generation through transcriptional up-regulation of a set of antioxidant enzymes such as CAT and CuZn-SOD [37]–[40]. Moreover, there is evidence demonstrating that some natural polyphenols, such as resveratrol, apigenin, carvacrol and humulon, can activate PPARs-dependent signaling [41]. This finding implies that PPARs are an important transcription factor in the GSE-regulatory signaling pathway leading to antioxidant enzymes.

Unlike the regulatory effects of GSE on the activities of both CAT and SOD, which are likely condition-dependent, GSE boosted GPx activity whether PMCs were pretreated with H_2_O_2_. In a previous study, the administration of proanthocyanidin extracted from grapeseeds also increased the GPx activity of rat heart under myocardial ischemic injury, but if the heart is not ischemic, increased GPx activity is not seen [36]. GPx has been proven to be regulated by diverse polyphenols via multiple approaches. For instance, β-naphthoflavone can bind and activate the aryl hydrocarbon receptor and subsequently induce the transcription of Nrf2, which is involved in the regulation of various antioxidant enzymes (i.e., GPx) [42]–[44]. In addition, flavonoids (i.e., cocoa flavonoids) can up-regulate GPx activity via the ERK1/2 signaling pathway [45]. This evidence suggests that GPx is regulated and controlled by GSE similarly to other polyphenols.

As to the reason why there was a partial divergence between GPx 1 mRNA expression and GPx activity, it was not surprising because the GPx superfamily, ignoring the members free from selenium, consists of at least four types of selenium-containing peroxidases that are encoded by separate genes [26]. GPx activity was tested as a whole for all GPx isoforms, while the mRNA of the GPx 1 gene was the only mRNA quantified in our trial. Thus, we could not rule out the possibility that other GPx genes might be upregulated by GSE and ultimately contribute to the increase in GPx enzymatic activity.

The increase of GPx activity in H_2_O_2_-pretreated PMCs incubated with GSE is believed to accelerate the depletion of intracellular GSH, as GSH provides a redox substrate for GPx as it implements H_2_O_2_ detoxification. However, GSH content in our study remained relatively stable. One possible explanation for this paradox is that accelerated consumption of GSH is accompanied by accelerated GSH generation. The depletion of cellular GSH could be opposed either by de novo synthesis or by reducing the GSSG formed (the oxidized form of GSH), and γ-glutamylcysteine synthetase (γ-GCS) and glutathione reductase (GRed), respectively, act as the key rate-limiting enzymes in these two process [46]. Several lines of evidence have shown that polyphenols (such as quercetin, epicatechin and epicatechin gallate) have the ability to increase GRed and γ-GCS activity [45], [47], [48], implying that GSE addition could make up for the increasing loss of GSH via the same mechanism in H_2_O_2_-pretreated PMCs of goats.

In summary, diverse concentrations of GSE strengthened TAOC to various extents in unstressed PMCs, and cell viability was enhanced by the addition of GSE within the dose range of 0.1 to 15 ug/ml after PMCs underwent H_2_O_2_ injury. In addition, GSE differentially regulated the mRNA expression levels and activities of CAT and SOD. The gene expression levels and activities of CAT and SOD were markedly low in unstressed PMCs with GSE addition at 10 ug/ml, while in H_2_O_2_-pretreated PMCs, GSE addition at the same dose induced a prominent increase in the expression levels and activities of CAT and SOD. Moreover, GSE addition enhanced GPx activity in both unstressed and stressed PMCs. These results suggest that GSE possesses the potential to improve antioxidant protection for ruminants by modulating antioxidant enzymes.
